# Integrative Nutritional Strategies for Performance, Recovery, and Weight Management in Taekwondo

**DOI:** 10.3390/nu18172894

**Published:** 2026-09-03

**Authors:** Adam Tawfiq Amawi, Walaa Jumah Alkasasbeh, Gerasimos V. Grivas, Parham Jalali, Aida Mohammadi

**Affiliations:** 1 Department of Movement Sciences and Sports Training, School of Sports Sciences, The University of Jordan, Amman 11942, Jordan; 2 Department of Physical Education, School of Sports Sciences, The University of Jordan, Amman 11942, Jordan; 3 Physical Education and Sports, Division of Humanities and Political Sciences, Hellenic Naval Academy, 18538 Pireas, Greece; 4 Wellness, Sport and Health Program, Department of Life Quality Studies, University of Bologna, Rimini Campus, 47921 Rimini, Italy; parham.jalali@studio.unibo.it; 5 Sport and Health Promotion Program, Department of Experimental Medicine and Surgery, University of Torvergata, 00133 Rome, Italy

**Keywords:** taekwondo, combat sports, sports nutrition, performance optimization, recovery, weight management, Relative Energy Deficiency in Sport (RED-S), sustainable athlete development

## Abstract

Taekwondo is a high-intensity, intermittent, weight-category combat sport requiring repeated explosive actions, rapid decision-making, and effective recovery between bouts. These demands, combined with congested competition schedules and weight-management requirements, make nutrition important for both performance and athlete health. This narrative review provides an evidence-informed synthesis of nutritional strategies relevant to taekwondo performance, recovery, and weight management by integrating available taekwondo-specific evidence with findings from comparable combat sports and established sports nutrition guidelines. Particular attention is given to energy and carbohydrate availability, protein intake, hydration and electrolyte balance, selected ergogenic aids, nutritional periodization, and safe weight management. The review also proposes an applied integrative framework illustrating how nutrition may influence interacting physiological, neuromuscular, cognitive, and recovery-related processes. A key limitation is the scarcity of taekwondo-specific intervention studies, meaning that several practical recommendations are necessarily extrapolated from comparable combat sports and broader athletic populations. From an applied perspective, nutritional priorities should include maintaining adequate energy and carbohydrate availability, supporting recovery through appropriate protein and fluid–electrolyte intake, and adopting gradual, individualized weight-management strategies that minimize dehydration, low energy availability, and Relative Energy Deficiency in Sport (RED-S) risk. Overall, nutritional strategies in taekwondo should be individualized and aligned with training load, competition demands, recovery needs, and weight-management goals. Further taekwondo-specific experimental studies are needed to refine these recommendations and strengthen sport-specific nutritional guidance.

## 1. Introduction

Taekwondo is a high-intensity, weight-category combat sport characterized by repeated explosive actions, short recovery periods, and substantial physiological and psychological demands [1]. Competitive performance depends on the interaction of anaerobic energy production, rapid force generation, neuromuscular coordination, technical execution, and cognitive decision-making under fatigue [2]. These demands are further amplified during tournaments, where athletes may be required to complete multiple bouts within a single day, often with limited time for recovery and physiological restoration [3].

Nutrition plays a central role in supporting performance, recovery, and competitive readiness in combat sports [4]. In taekwondo, nutritional strategies must address the energetic and metabolic requirements of high-intensity intermittent exercise while also considering the specific challenges imposed by weight-category competition. Practices such as rapid weight loss, dehydration, and severe energy restriction remain common among taekwondo athletes [5], despite evidence linking these behaviours to impaired performance, delayed recovery, increased injury risk, and adverse psychological outcomes [6].

Current sport nutrition guidelines provide strong evidence for the importance of carbohydrate availability, protein intake, hydration, and selected ergogenic aids in optimizing exercise performance and recovery [7,8]. However, much of this evidence is derived from endurance sports, team sports, or general athletic populations, with comparatively limited synthesis focused specifically on taekwondo [9]. As a result, coaches and athletes may rely on generalized or anecdotal nutritional approaches that do not fully reflect the physiological, cognitive, and practical demands of taekwondo competition [10,11,12].

Previous reviews and position statements in combat-sport nutrition have addressed several important areas, including energy and macronutrient requirements, hydration and rehydration, dietary supplementation, recovery, and rapid weight-loss practices [13]. More recent reviews have also examined specific aspects of nutrition in taekwondo, particularly dietary supplementation and its effects on physical performance, recovery, and other athlete-related outcomes [14]. However, much of the available evidence has been synthesized across heterogeneous combat-sport populations [15], despite evidence of sport-specific differences in body composition and nutritional intake among combat disciplines [16]. The present review addresses this gap by bringing these domains together within an applied, evidence-informed framework and by explicitly distinguishing taekwondo-specific evidence from recommendations extrapolated from comparable combat sports and broader athletic populations.

Therefore, this narrative review aims to provide an evidence-informed synthesis of nutritional strategies relevant to performance, recovery, and weight management in taekwondo athletes by integrating the available taekwondo-specific evidence with findings from comparable combat sports and established sports nutrition guidelines where direct taekwondo-specific evidence is limited. Specifically, the review examines the roles of carbohydrate availability, protein intake, hydration and electrolyte balance, selected ergogenic aids, nutritional periodization, and individualized approaches across training, competition, and recovery phases, while explicitly distinguishing recommendations supported by taekwondo-specific studies from those extrapolated from broader athletic populations.

In addition, this review proposes a taekwondo-specific applied integrative framework that organizes current evidence into a practical perspective for nutritional decision-making. Rather than introducing a new theoretical model or presenting validated taekwondo-specific recommendations, the framework synthesizes the best available evidence to illustrate how nutrition may influence interacting physiological, neuromuscular, cognitive, and recovery processes within the context of taekwondo. This evidence-informed approach is intended to support individualized practice while highlighting important areas where future taekwondo-specific research is needed.

## 2. Methodology

This narrative review was conducted in accordance with the principles of the Scale for the Assessment of Narrative Review Articles (SANRA) to enhance transparency and reporting quality [17]. A structured literature search was conducted in PubMed, Scopus, Web of Science, and Google Scholar, with the final search performed on 15 March 2026. The search combined terms related to the sport and the main nutritional domains addressed in the review using Boolean operators. The core search string was: (“taekwondo” OR “combat sport”) AND (“sports nutrition” OR nutrition OR “energy availability” OR carbohydrate OR protein OR hydration OR electrolyte OR recovery OR “ergogenic aid” OR caffeine OR creatine OR “beta-alanine” OR “weight management” OR “rapid weight loss” OR “low energy availability” OR “Relative Energy Deficiency in Sport” OR “RED-S”). The syntax was adapted where necessary for each database, and reference lists of relevant reviews, consensus statements, and position stands were additionally screened for pertinent literature.

Eligible sources included peer-reviewed systematic reviews, meta-analyses, experimental and observational studies, consensus statements, position stands, and established sports-nutrition guidelines addressing performance, recovery, cognitive function, nutritional periodization, or weight management. Taekwondo-specific studies were prioritized. When direct evidence was limited, evidence from comparable combat sports was considered, followed, where necessary, by evidence from broader athletic populations and established sports-nutrition guidance. Studies were excluded when they were not relevant to the nutritional domains addressed in this review or lacked meaningful applicability to athletic performance, recovery, or weight management.

As this was a narrative review, study selection was documented descriptively rather than through a PRISMA-style systematic-review flow diagram. Records were screened by title and abstract, followed by full-text consideration of potentially relevant publications. However, numerical counts of records identified, duplicates removed, and articles excluded at each stage were not prospectively recorded and are therefore not reported. The selected evidence was subsequently organized according to the principal domains of the review, including energy and carbohydrate availability, protein intake, hydration and electrolyte balance, ergogenic aids, nutritional periodization, cognitive and psychological performance, recovery, individualized nutrition, and weight management.

Evidence was synthesized narratively according to its source, directness, consistency, and applicability to taekwondo. Studies conducted specifically in taekwondo athletes were considered direct sport-specific evidence. Evidence from comparable combat sports was considered indirect but contextually relevant, whereas evidence from broader athletic populations was used primarily to support established sports-nutrition principles when taekwondo-specific evidence was limited. Greater interpretive weight was given to systematic reviews, meta-analyses, controlled intervention studies, and authoritative consensus or position statements, while findings from observational studies and recommendations based predominantly on indirect evidence were interpreted more cautiously.

No formal risk-of-bias assessment or certainty-of-evidence grading system was applied. Accordingly, descriptors used throughout the manuscript refer to the source and directness of the evidence rather than to formally graded methodological quality or certainty. Recommendations supported mainly by indirect or limited evidence were therefore phrased cautiously and explicitly identified as requiring further taekwondo-specific investigation. This approach reflects the evidence-informed narrative design of the review and should not be interpreted as a formal systematic assessment or grading of the evidence.

## 3. Evidence-Informed Conceptual Synthesis of Nutritional Modulation in Taekwondo

Performance in taekwondo emerges from the interaction of physiological, neuromuscular, cognitive, and recovery-related processes, all of which may be influenced by nutritional status [9,18]. Within this context, nutrition should not be considered an isolated determinant of performance but rather a central modulator of multiple interconnected systems. This evidence-informed conceptual synthesis organizes the available evidence to illustrate how nutritional factors may influence physiological readiness, cognitive function, between-bout recovery, and weight-management outcomes in taekwondo athletes [19,20]. It draws on taekwondo-specific evidence where available and uses evidence from comparable combat sports and established sports nutrition guidelines where direct sport-specific evidence is limited.

At the physiological level, adequate energy availability and macronutrient intake support the metabolic demands of high-intensity intermittent exercise [21,22]. Carbohydrate availability is particularly important for sustaining glycolytic energy production and delaying fatigue during repeated explosive actions [23], although much of the supporting evidence is derived from broader sports nutrition literature and comparable intermittent sports. Protein intake contributes to muscle repair, training adaptation, and the preservation of lean body mass, especially during periods of energy restriction or weight management [24]; however, evidence specific to taekwondo athletes remains limited. Hydration status also plays a critical role by supporting cardiovascular stability, thermoregulation, and neuromuscular function, which are essential for maintaining performance across multiple bouts within a competition day [25,26]. Collectively, these relationships are supported by varying combinations of taekwondo-specific, combat-sport, and broader sports-nutrition evidence.

Decision-making speed, reaction time, attentional focus, and perceived exertion can be affected by glucose availability, hydration status, and selected ergogenic aids [18,27], although direct taekwondo-specific evidence for these outcomes remains limited. Given the rapid and unpredictable nature of taekwondo combat, such cognitive impairments could plausibly affect tactical execution and technical accuracy; however, their direct performance relevance requires further investigation in taekwondo athletes.

Recovery is another key element of this conceptual synthesis, particularly in tournaments involving congested schedules and limited time between bouts [28]. Nutritional strategies implemented between bouts and after competition, including carbohydrate replenishment, protein intake, and fluid-electrolyte restoration, may accelerate glycogen resynthesis, support muscle repair, and help maintain neuromuscular performance [29,30]; however, few intervention studies have specifically evaluated these strategies in taekwondo athletes. Inadequate recovery nutrition may contribute to cumulative fatigue, impaired subsequent performance, and increased injury risk [31,32].

In weight-category sports such as taekwondo, nutritional modulation must also be considered in relation to weight-management practices [33]. Strategies that compromise energy availability or hydration status may disrupt physiological and cognitive function, thereby impairing performance and increasing the risk of Relative Energy Deficiency in Sport (RED-S) [34]. This relationship is supported primarily by evidence from combat sports and broader athletic populations. Therefore, performance-oriented nutrition should be integrated with safe, gradual, and sustainable weight-management strategies.

Accordingly, this conceptual synthesis should be interpreted as an applied organization of existing sports-nutrition evidence within the competitive context of taekwondo, rather than as a novel theoretical model or a validated taekwondo-specific framework. Its purpose is to contextualize current evidence in relation to repeated bouts, limited recovery periods, weight-category requirements, and cognitive demands under fatigue, while identifying areas in which direct taekwondo-specific evidence remains insufficient.

## 4. Physiological and Metabolic Demands of Taekwondo

Taekwondo is a high-intensity intermittent combat sport characterized by repeated explosive actions, rapid accelerations and decelerations, short recovery periods, and frequent changes in movement direction [35]. Competitive performance requires the integration of technical skill, tactical decision-making, neuromuscular power, and the capacity to sustain repeated high-intensity efforts across rounds and bouts.

From a metabolic perspective, taekwondo performance is supported by the interaction of both anaerobic and aerobic energy systems. The phosphagen (ATP–PCr) system contributes predominantly to brief maximal actions, such as explosive kicks, rapid attacks, and defensive movements, whereas anaerobic glycolysis supports repeated high-intensity exchanges within rounds [36,37]. Although taekwondo is often described as an anaerobically demanding sport, aerobic metabolism also plays an important role in phosphocreatine resynthesis, between-exchange recovery, and the maintenance of performance across repeated bouts during tournament competition.

These metabolic demands may result in phosphocreatine depletion, elevated lactate accumulation, metabolic acidosis, and progressive neuromuscular fatigue, particularly when athletes compete multiple times within a single day [38]. Therefore, nutritional strategies that support carbohydrate availability, fluid-electrolyte balance, and rapid recovery are essential for maintaining repeated-effort capacity and delaying fatigue.

In addition to metabolic stress, taekwondo imposes substantial neuromuscular and cognitive demands. Athletes must generate high levels of force rapidly, maintain postural control, react to unpredictable opponent actions, and make accurate tactical decisions under fatigue [39]. Hydration status is especially relevant in this context, as mild dehydration, often associated with weight-cutting practices, may impair power output, reaction time, cognitive function, and perceived exertion [40]. Most evidence supporting these effects is derived from combat sports and broader athletic populations, with comparatively fewer studies conducted specifically in taekwondo athletes. Together, these physiological, metabolic, neuromuscular, and cognitive characteristics provide the rationale for the evidence-informed nutritional strategies discussed in the following sections, while recognizing that some recommendations are based on indirect evidence where taekwondo-specific research remains limited. Given the limited availability of taekwondo-specific nutrition research, the evidence supporting the nutritional topics discussed in this review varies in both source and directness. Table 1 summarizes the primary sources of evidence for each major nutritional domain, distinguishing between direct taekwondo-specific evidence and evidence derived from comparable combat sports, broader athletic populations, and established sports nutrition guidelines and consensus statements.

## 5. Nutritional Interventions to Optimize Performance

### 5.1. Carbohydrate Availability

Adequate carbohydrate availability is considered important for supporting repeated high-intensity efforts in taekwondo, where athletes perform rapid and explosive actions across multiple rounds and bouts [41]. This interpretation is supported by available taekwondo-specific evidence together with findings from comparable combat sports and established sports-nutrition guidelines. Muscle glycogen contributes substantially to anaerobic energy production during repeated high-intensity exchanges and may help delay fatigue, particularly during congested competition schedules [44]. Conversely, low carbohydrate availability may accelerate fatigue and reduce the ability to maintain power output and technical effectiveness during repeated efforts [45].

Carbohydrate timing may be particularly relevant on competition days, when athletes may be required to perform several bouts with limited recovery time [46]. Beyond its metabolic role, adequate carbohydrate availability may also support cognitive function, perceived exertion regulation, and decision-making accuracy, all of which are important for tactical performance in taekwondo [18]. However, direct taekwondo-specific intervention evidence remains limited; therefore, competition-day carbohydrate strategies should be interpreted as contextually relevant recommendations derived partly from comparable intermittent sports and established sports-nutrition guidance rather than as validated taekwondo-specific protocols.

### 5.2. Protein Intake

Protein intake is generally considered important for muscle recovery, repair, and adaptation in taekwondo athletes [14], although much of the supporting evidence is derived from broader athletic populations. Given the repeated neuromuscular stress imposed by kicking, rapid directional changes, and high-intensity combat actions, adequate protein intake may support muscle protein synthesis and attenuate exercise-induced muscle damage [47]. This is particularly relevant during periods of intensified training, competition, or incomplete recovery between sessions and bouts [8].

In weight-category sports, protein intake may also contribute to preserving lean body mass during periods of energy restriction or gradual weight reduction [8]. Distributing protein intake evenly across the day, including post-exercise intake, may enhance recovery and support the maintenance of muscle mass without compromising weight-management goals [48]. However, the optimal application of these strategies specifically in taekwondo remains insufficiently investigated. Protein strategies should therefore be individualized according to training demands, energy availability, and weight-management goals.

### 5.3. Hydration and Electrolyte Balance

Hydration status is considered an important determinant of both physical and cognitive performance in taekwondo [5,43], although the supporting evidence is derived largely from combat-sport and broader sports-nutrition literature. Dehydration may impair power output, reaction time, decision-making, and perceived exertione [49]. These effects are particularly relevant during tournaments involving multiple bouts, short recovery periods, and potential exposure to heat stress or weight-cutting practices.

Rapid weight-loss strategies may further exacerbate dehydration and electrolyte disturbances, increasing the risk of performance decrements, delayed recovery, and adverse health outcomes [50]. Fluid and electrolyte replacement, including sodium, is generally recommended to facilitate rehydration and restoration of fluid balance [51,52]. However, specific post-weigh-in and between-bout rehydration protocols have not been sufficiently validated in taekwondo athletes. Accordingly, their application should be individualized according to the magnitude of fluid loss, recovery time available, environmental conditions, and athlete tolerance.

### 5.4. Ergogenic Aids

Selected ergogenic aids may offer potential performance benefits for taekwondo athletes; however, the directness of evidence varies considerably across supplements, and findings should not be assumed to transfer uniformly to taekwondo. Caffeine has been investigated more directly in taekwondo and may enhance selected aspects of alertness and high-intensity performance [53].

More recently, Delleli et al. [54] reported in a double-blind, randomized crossover trial that low-dose caffeine combined with warm-up music improved several technical–tactical and perceptual outcomes during simulated taekwondo combat in male athletes. However, because the intervention combined caffeine with music, these findings should not be interpreted as evidence of an independent caffeine effect. Individual tolerance, habitual intake, timing, and potential adverse effects should also be considered. Evidence supporting creatine and beta-alanine specifically in taekwondo is more limited and is derived largely from broader athletic or combat-sport populations. Creatine may theoretically support repeated high-intensity efforts and explosive performance [55], changes in body mass. Similarly, beta-alanine may be relevant because of its established role in increasing intramuscular buffering capacity during high-intensity exercise [56], but its performance benefits should not be assumed to be taekwondo-specific without direct intervention evidence.

Overall, ergogenic aids should be considered individualized adjuncts rather than established taekwondo-specific recommendations, with decisions guided by the strength and directness of evidence for each supplement, athlete tolerance, competitive demands, and body-mass goals.

## 6. Nutritional Periodization in Taekwondo Athletes

Nutritional periodization refers to the strategic adjustment of dietary intake according to the specific demands of training, competition, recovery, and body-composition goals [57]. Rather than applying fixed dietary recommendations across all phases, athletes may benefit from adapting energy intake, macronutrient distribution, hydration strategies, and supplement use according to training load, competition schedule, and individual needs [8,58]. In taekwondo, this approach is particularly relevant because athletes must simultaneously support high-intensity intermittent performance, rapid recovery, cognitive readiness, and safe weight management within the constraints of weight-category competition [15,59,60]. Accordingly, this section focuses on the phase-specific application of the nutritional principles discussed previously rather than reiterating their underlying physiological rationale.

### 6.1. Training Phase

During the training phase, nutritional strategies should primarily support training quality, physiological adaptation, and recovery between sessions [61]. Adequate energy availability is essential to sustain repeated high-intensity training, prevent excessive fatigue, and reduce the risk of low energy availability [21]. Macronutrient intake should therefore be adjusted according to daily training demands [62,63]. In selected situations, strategies such as training with reduced carbohydrate availability may be used to stimulate metabolic adaptations [64]. However, their application in taekwondo should be approached cautiously because technical quality, explosive power, and high-intensity output may be compromised when carbohydrate availability is insufficient. Therefore, the primary nutritional goal during the training phase should be to maximize training quality, support adaptation, and minimize unnecessary fatigue accumulation.

### 6.2. Pre-Competition Phase

The pre-competition phase should focus on maximizing physiological and cognitive readiness while ensuring that athletes meet their required weight category safely [65,66]. Carbohydrate availability, hydration, and food choices should be adjusted to competition demands and individual tolerance, while gastrointestinal discomfort should be minimized [67,68]. When body-mass reduction is required, gradual and controlled strategies should be prioritized over aggressive energy restriction or dehydration [13]. Selected ergogenic aids intended for competition should be tested during training before use.

### 6.3. Competition Day

Competition day in taekwondo often involves multiple bouts within a relatively short time frame, requiring practical and rapidly tolerated nutritional strategies [69]. Carbohydrate and fluid–electrolyte intake should therefore be individualized according to weigh-in timing, recovery intervals, sweat losses, environmental conditions, and gastrointestinal tolerance [70,71,72]. Where appropriate, previously tested caffeine strategies may also be used, with consideration of dose, timing, tolerance, and potential side effects [53]. Following weigh-in, priority should be given to rehydration and easily digestible carbohydrate intake according to the magnitude of body-mass loss and the time available before the first bout. Between bouts, nutritional intake should be adapted to the recovery interval, with rapidly tolerated carbohydrate sources favored when recovery time is short and light carbohydrate–protein options considered when longer intervals permit [73,74]. Familiar and portable foods and fluids should be prepared in advance when access at competition venues is limited.

### 6.4. Recovery Phase

Recovery nutrition should prioritize glycogen restoration, muscle repair, and rehydration through appropriate carbohydrate, protein, and fluid–electrolyte intake [75,76,77]. When recovery intervals are short, early nutrient intake may be particularly relevant [78]. In tournament settings, practical foods and fluids should therefore be planned in advance to facilitate recovery between repeated competitive efforts. Overall, nutritional periodization in taekwondo involves adapting established nutritional principles to training load, competition timing, recovery opportunities, and weight-management demands rather than applying fixed recommendations across all phases.

## 7. Nutritional Influences on Cognitive and Psychological Performance

In taekwondo, competitive performance is not determined solely by physical capacity but also by cognitive and psychological processes that support effective technical and tactical execution under dynamic and high-pressure conditions [79]. Athletes must rapidly process visual information, anticipate opponents’ actions, select appropriate responses, and execute precise motor skills, often while experiencing fatigue [80]. Therefore, nutritional status may influence performance not only through metabolic and neuromuscular pathways but also through its effects on cognitive function, perceived exertion, and psychological readiness [34], although much of the available evidence is derived from broader exercise cognition and combat-sport literature rather than taekwondo-specific intervention studies.

### 7.1. Reaction Time

Reaction time is an important determinant of success in taekwondo, where small temporal differences may influence the effectiveness of offensive and defensive actions [81,82]. Nutritional factors such as carbohydrate availability and hydration status may affect neural processing, neuromuscular coordination, and response speed [83,84]. Adequate carbohydrate intake helps maintain blood glucose availability, which is important for brain function, particularly during prolonged or repeated high-intensity efforts [85]. Hydration status is also relevant, as even mild dehydration may impair neuromuscular coordination, reaction time, and taekwondo-specific performance [43,86,87]. In addition, caffeine may acutely enhance alertness and reaction time, although responses vary according to dose, timing, habitual caffeine intake, and individual tolerance [53]. These considerations suggest that maintaining adequate fueling and hydration may help preserve rapid response capacity during competition. However, direct taekwondo-specific intervention studies evaluating these nutritional effects on reaction time remain limited.

### 7.2. Decision-Making

Effective decision-making in taekwondo requires rapid evaluation of opponents’ movements and selection of appropriate tactical responses under time pressure [88,89]. Nutritional status may influence cognitive flexibility, attention, and executive function, which are important for decision-making during complex and unpredictable combat situations [90,91,92]. Low energy availability, inadequate carbohydrate intake, or dehydration may impair cognitive performance, potentially leading to slower or less accurate decisions [93,94]. Conversely, maintaining adequate energy availability and hydration may support brain function and help athletes respond more effectively to dynamic competitive demands [95]. Although direct evidence in taekwondo remains limited, these mechanisms may be relevant given the sport’s dependence on rapid perception–action coupling; however, their direct applicability to taekwondo performance requires further investigation.

### 7.3. Focus and Attention

Sustained attention and concentration are essential for maintaining technical accuracy and tactical awareness across multiple bouts in a competition setting [96]. Adequate nutritional support may contribute to attentional control and help reduce mental fatigue during competition [83,97,98].

This is especially important when athletes are exposed to repeated bouts, psychological pressure, and limited recovery time. Caffeine supplementation has been reported to enhance concentration, vigilance, and perceived energy, which may be advantageous in high-intensity combat sports [99,100,101]. However, excessive intake may produce undesirable effects, including anxiety, gastrointestinal discomfort, sleep disturbance, or impaired fine motor control. Therefore, caffeine use should be individualized and tested during training before being applied in competition. Current recommendations are based primarily on broader sports nutrition evidence rather than taekwondo-specific intervention studies.

### 7.4. Perception of Fatigue

Perceived exertion and mental fatigue can influence performance regulation in taekwondo, particularly during repeated high-intensity exchanges and tournament formats [102]. Nutritional status may modulate perceived effort, with adequate carbohydrate availability contributing to the maintenance of exercise intensity and delaying fatigue-related performance decline [23]. Low energy availability and dehydration may increase perceived exertion, reduce motivation, and negatively affect psychological readiness [95]. In contrast, appropriate nutritional strategies may help attenuate fatigue perception, allowing athletes to sustain high-intensity efforts and maintain tactical effectiveness throughout competition [103].

Overall, nutrition may be considered a potential modulator of cognitive and psychological performance in taekwondo, in addition to its established role in supporting physiological function, although direct taekwondo-specific evidence remains limited. Strategies that maintain energy availability, hydration, and appropriate use of ergogenic aids may help preserve reaction time, decision-making, focus, and fatigue regulation, especially in high-level competition where performance margins are small, although many of these recommendations are extrapolated from comparable athletic populations.

## 8. Nutrition and Recovery in Taekwondo

Effective recovery nutrition is essential for taekwondo athletes, particularly in tournament settings where multiple bouts may be performed within a short period of time [42]. Rather than reiterating the individual roles of carbohydrate, protein, and fluid–electrolyte intake described in the preceding sections, recovery nutrition should be considered in relation to the limited recovery intervals and repeated-bout demands characteristic of taekwondo competition. High-intensity combat efforts may result in glycogen depletion, neuromuscular fatigue, muscle damage, and fluid loss, making the timing and practical implementation of recovery strategies particularly important when subsequent bouts or training sessions follow within a short period [8,78].

Recovery nutrition may also contribute to the regulation of exercise-induced inflammation and muscle soreness, thereby supporting readiness for subsequent training sessions or matches [104]. In congested tournament schedules, inadequate recovery nutrition may lead to cumulative fatigue, reduced technical accuracy, impaired decision-making, and increased injury risk [105]. Accordingly, recovery strategies in taekwondo should be practical, individualized, and timed according to the interval between bouts, the magnitude of body-mass loss, gastrointestinal tolerance, and the athlete’s overall competition schedule.

## 9. Individual Variability and Personalized Nutrition Strategies

Optimal nutritional strategies in taekwondo cannot be universally applied, as athletes differ substantially in physiological responses, metabolic demands, training status, body composition, weight-management requirements, and tolerance to specific foods or supplements [9,106]. Personalized nutrition approaches are therefore essential to maximize performance, support recovery, and reduce the health risks associated with inappropriate or overly standardized dietary practices [107,108]. These principles are supported by available taekwondo-specific evidence together with broader sports nutrition research where direct taekwondo-specific evidence is limited.

### 9.1. Sex Differences

Sex-specific physiological and hormonal characteristics may influence nutritional requirements and responses in taekwondo athletes [18]. Female athletes may be particularly vulnerable to low energy availability and Relative Energy Deficiency in Sport (RED-S), especially in weight-category sports where pressure to achieve a specific body mass is common [109]. Hormonal fluctuations across the menstrual cycle may also influence substrate utilization, hydration status, thermoregulation, and perceived exertion [110,111]. Most evidence describing these physiological responses is derived from broader exercise physiology and sports nutrition research rather than taekwondo-specific studies. Although practical recommendations should remain individualized, these factors suggest that female athletes may require closer monitoring during phases of intensified training, weight management, or competition preparation [112,113]. Male athletes may present different body-composition goals, hormonal responses, and energy requirements, indicating that nutritional strategies should also be tailored according to training demands, body-mass goals, and individual physiological responses [114]. Further taekwondo-specific studies are needed to better define sex-specific nutritional recommendations.

### 9.2. Athlete Level (Training Status)

Nutritional requirements vary according to training status, competitive level, and competition frequency [8]. Current recommendations are informed by available taekwondo-specific evidence together with established sports nutrition guidelines and findings from comparable athletic populations. Elite taekwondo athletes typically experience higher training loads, greater performance demands, and more congested competition schedules, which may require more precise strategies for carbohydrate availability, hydration, recovery nutrition, and supplement use [8,115]. In contrast, novice or developing athletes may benefit most from consistent adherence to fundamental nutritional principles, including adequate total energy intake, regular meal timing, sufficient protein intake, and appropriate hydration. Advanced athletes may also benefit from more sophisticated strategies such as targeted carbohydrate periodization, individualized competition-day fueling, and strategic use of ergogenic aids [20,116], although direct taekwondo-specific evidence supporting these advanced strategies remains limited. However, these approaches should be implemented only when the athlete has already established sound dietary habits and has tested specific strategies during training. Nutritional planning should therefore be aligned with the athlete’s developmental stage, training intensity, competition schedule, and practical experience.

### 9.3. Body Weight and Composition

Body weight and composition are central considerations in taekwondo due to the sport’s weight-category structure [117]. Nutritional strategies should support an optimal balance between competitive weight, lean body mass, power production, and recovery capacity [58]. These recommendations are supported by available evidence from taekwondo, comparable combat sports, and established sports nutrition guidelines.

Athletes with greater lean body mass may require higher absolute protein intake to support muscle maintenance and repair [118], whereas athletes aiming to reduce body mass must balance energy restriction with adequate nutrient intake to minimize performance decrements and health risks [119]. Gradual and periodized weight-management strategies are preferable to rapid weight-loss approaches, as they may help preserve lean mass, maintain metabolic function, reduce dehydration risk, and lower the likelihood of RED-S [106]. Body-composition goals should therefore be planned across the training cycle rather than addressed only in the final days before competition. This approach may allow athletes to reach their target weight while maintaining training quality, cognitive function, and recovery capacity, although further taekwondo-specific intervention studies are needed to optimize body-composition strategies across different competitive levels.

### 9.4. Individual Response to Nutritional Interventions

Athletes differ in their responses to nutritional strategies, including carbohydrate intake, hydration practices, caffeine, creatine, beta-alanine, and other ergogenic aids [8,120]. For example, the ergogenic effects of caffeine may vary according to genetic factors, habitual intake, dose, timing, and individual tolerance [120]. Similarly, gastrointestinal tolerance to foods, fluids, and supplements may strongly influence the practicality of competition-day nutrition, particularly when recovery time between bouts is limited [121,122]. Personalized nutrition should therefore combine objective monitoring with athlete feedback. Practical indicators may include body mass, body composition, hydration status, perceived exertion, gastrointestinal symptoms, recovery markers, training quality, and subjective well-being [123]. Integrating these data with the athlete’s preferences and competition demands may support more adaptive, athlete-centered decision-making [124], although evidence describing individualized nutritional responses in taekwondo athletes specifically remains limited.

Overall, individualized nutrition in taekwondo requires moving beyond generalized guidelines toward context-specific strategies that reflect each athlete’s sex, training status, body-composition goals, weight-management history, and response to nutritional interventions. Such an approach may improve performance consistency, optimize recovery, and reduce health risks associated with inappropriate or standardized dietary practices. This personalized approach is informed by the best available taekwondo-specific evidence together with broader sports nutrition research, while highlighting the need for further sport-specific studies to refine individualized recommendations.

## 10. Weight Management Challenges in Taekwondo

Weight management represents one of the most important nutritional challenges in taekwondo because athletes compete within defined weight categories [125]. Alternative categorization approaches have also been proposed. Bešlija et al. [126] suggested a height-based model for combat sports that may reduce anthropometric disparities and potentially limit harmful body-weight manipulation. Although achieving an appropriate competitive body mass may provide tactical or anthropometric advantages, inappropriate weight-management practices can compromise both performance and health. Rapid weight loss, acute dehydration, severe energy restriction, and repeated weight cycling remain common concerns in combat sports and may be particularly problematic when athletes are required to compete multiple times within a short period. Rapid weight-loss practices and chronic low energy availability may increase the risk of Relative Energy Deficiency in Sport (RED-S), particularly when athletes repeatedly restrict energy intake or use dehydration-based strategies to achieve a target weight [127]. These practices may impair metabolic function, hormonal regulation, bone health, immune function, mood, concentration, and competitive performance [128,129,130,131,132,133,134]. In taekwondo, such effects are especially relevant because athletes must maintain explosive power, rapid decision-making, reaction time, technical accuracy, and recovery capacity across repeated bouts.

Evidence increasingly supports gradual, periodized weight-management strategies as safer and more effective alternatives to extreme weight-cutting [135]. Rather than focusing on acute body-mass reduction immediately before competition, athletes should aim to manage body composition across the training cycle. This approach allows sufficient time to preserve lean mass, maintain energy availability, support training quality, and reduce the need for aggressive dehydration practices. Adequate protein intake, appropriate carbohydrate availability, hydration maintenance, and regular monitoring of body mass and body composition should form the basis of this strategy [136]. Effective weight management in taekwondo should therefore prioritize long-term performance sustainability and athlete health over short-term reductions in body mass. Coaches, athletes, and support staff should discourage extreme weight-cutting practices and instead implement structured, individualized, and evidence-based plans that account for training load, competition schedule, sex, maturation status, weight history, and recovery needs.

When body-mass reduction is required, weight-loss strategies should begin sufficiently in advance of competition to allow gradual reductions in body mass while minimizing the need for aggressive weight-cutting during the final days before the official weigh-in. Throughout the weight-management period, athletes should be monitored regularly to ensure that performance, recovery, and health are maintained [134,135]. Practical monitoring may include periodic assessment of body mass, body composition, hydration status, training quality, recovery, and subjective well-being [137].

Because prolonged or excessive energy restriction may increase the risk of Relative Energy Deficiency in Sport (RED-S) [138], practitioners should also monitor for early indicators of low energy availability, including excessive fatigue, impaired recovery, recurrent illness, unexpected declines in performance, mood disturbances, and, where appropriate, menstrual dysfunction in female athletes [139,140]. When available, validated screening tools such as the IOC Relative Energy Deficiency in Sport Clinical Assessment Tool (RED-S CAT2) may assist practitioners in identifying athletes at increased risk and guiding appropriate management strategies [141].

**Figure 1:**
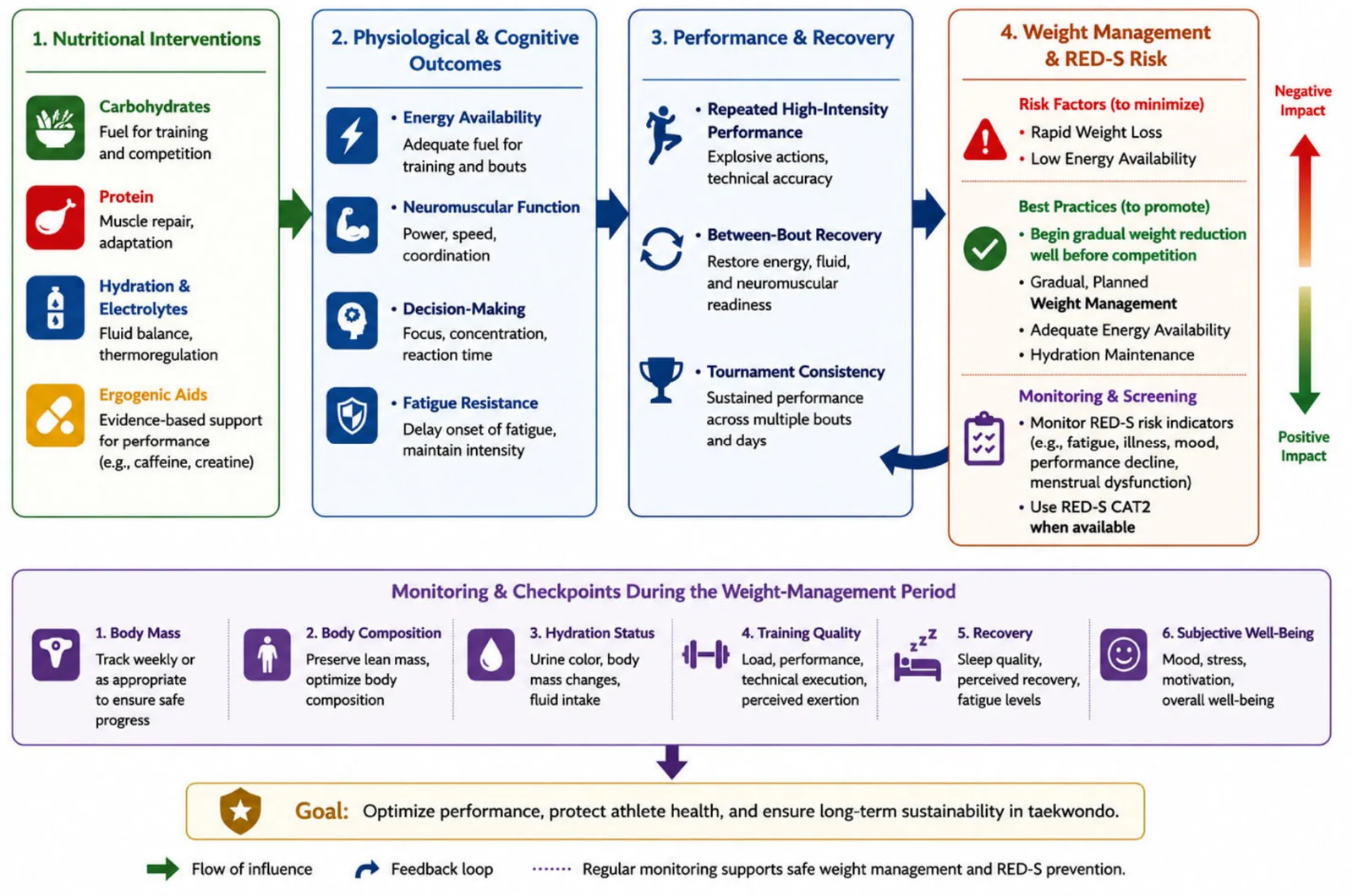
Evidence-Informed Conceptual Synthesis of Nutrition, Performance, Recovery, and Safe Weight Management in Taekwondo. Note: The framework represents an applied, evidence-informed decision-support tool and should not be interpreted as a validated taekwondo-specific clinical guideline. RED-S, Relative Energy Deficiency in Sport; RED-S CAT2, Relative Energy Deficiency in Sport Clinical Assessment Tool Version 2.

Figure 1 presents an evidence-informed conceptual framework designed to support nutritional decision-making in taekwondo. The framework integrates the best available taekwondo-specific evidence with findings from comparable combat sports, broader athletic populations, and established sports nutrition guidelines. It should therefore be interpreted as an applied decision-support tool rather than a validated taekwondo-specific clinical guideline.

## 11. Discussion

This narrative review indicates that nutritional strategies relevant to taekwondo should be interpreted within the specific demands of a high-intensity, weight-category combat sport, while recognizing substantial variation in the directness of the available evidence. Taekwondo-specific evidence is available for selected aspects of carbohydrate availability, hydration, supplementation, dietary intake, and weight-management practices [5,9,14,41,42,43]. However, several recommendations concerning protein intake, recovery nutrition, nutritional periodization, and individualized strategies remain informed largely by evidence from comparable combat sports and broader athletic populations [8,13,57,75,76]. This distinction is important because established sports-nutrition principles should not automatically be interpreted as validated taekwondo-specific protocols.

Among the areas supported by direct sport-specific evidence, carbohydrate availability and hydration appear particularly relevant. Hsu et al. [41] demonstrated the importance of carbohydrate restoration following energy restriction in taekwondo athletes, whereas Zheng et al. [43] showed that hydration status can influence cognitive function and taekwondo-specific kicking performance. These findings are especially relevant given the repeated high-intensity actions and cognitive–technical demands of competition. Nevertheless, optimal nutrient timing, dosing, and between-bout strategies remain insufficiently established specifically for taekwondo, and recommendations in these areas still rely partly on broader sports nutrition evidence [44,57,67,78].

Weight management represents a particularly important consideration because nutritional strategies intended to achieve competitive body mass may conflict with adequate energy availability and hydration. Dehydration-based pre-competition weight-loss practices have been documented in taekwondo athletes [5], while broader evidence from weight-category sports associates aggressive weight loss with adverse physiological, performance, and health consequences [129,134,142]. Repeated or prolonged energy restriction may also increase the risk of low energy availability and RED-S [127,138,140]. These findings support prioritizing gradual and periodized weight-management approaches that minimize unnecessary dehydration and preserve nutritional support for training, recovery, and competition.

The evidence-informed conceptual synthesis presented in this review should therefore be understood as an applied organization of existing evidence rather than a novel theoretical model or a validated taekwondo-specific framework. Its value lies in contextualizing nutritional principles around the interacting demands of repeated high-intensity performance, cognitive–technical execution, between-bout recovery, and weight management. This approach also highlights an important limitation of the current literature: physiological plausibility and evidence from other athletic populations are considerably stronger than direct intervention evidence for several nutritional strategies in taekwondo.

The narrative design of this review should also be considered when interpreting these findings. No formal risk-of-bias assessment or certainty-of-evidence grading was undertaken, and the narrative selection and synthesis of heterogeneous evidence may be more susceptible to selection and interpretive bias than a systematic review. Accordingly, the findings should be interpreted as an evidence-informed synthesis of the best available taekwondo-specific and indirect evidence rather than as definitive or validated taekwondo-specific nutritional recommendations. Future research should prioritize high-quality controlled and longitudinal studies conducted in taekwondo athletes under ecologically relevant training and competition conditions. Particular attention is needed to competition-day fueling, post-weigh-in rehydration, between-bout recovery, nutrient timing, and the longer-term consequences of repeated weight cycling and low energy availability. Studies across different sexes, competitive levels, and weight categories that integrate physiological, cognitive, technical-performance, recovery, and health outcomes would help determine whether currently recommended strategies translate into meaningful sport-specific benefits.

## 12. Practical Applications and Recommendations

From an applied perspective, nutrition in taekwondo should be integrated into the overall training, competition, recovery, and weight-management plan rather than treated as a separate component [143,144]. Nutritional planning should be individualized according to training load, competition schedule, body-composition goals, weight-category requirements, gastrointestinal tolerance, and athlete-specific responses [144]. Effective implementation should also include nutrition education for athletes and coaches, particularly regarding adequate energy availability, safe weight management, competition-day fueling, and the importance of testing nutritional strategies during training before their use in competition [145].

During training, nutritional strategies should support energy availability, adaptation, and recovery [146], whereas pre-competition planning should prioritize competitive readiness while minimizing reliance on aggressive weight-cutting practices [147]. On competition day, strategies should account for weigh-in timing, the number of bouts, recovery intervals, gastrointestinal tolerance, and access to suitable foods and fluids. Familiar, portable, and easily digestible carbohydrate-containing foods and appropriate fluids may facilitate fueling and hydration across repeated bouts [148]. Ergogenic aids, when used, should be individualized, tested during training, and implemented in accordance with sport regulations and anti-doping requirements.

Particular attention should be given to weight management and athlete monitoring. Gradual and periodized approaches should be prioritized, with monitoring of body mass, body composition, hydration status, recovery, training quality, and indicators of low energy availability and RED-S [149,150]. Practical barriers, including travel, limited food availability at competition venues, time constraints, and gastrointestinal discomfort, should also be considered when developing individualized plans.

**Figure 2:**
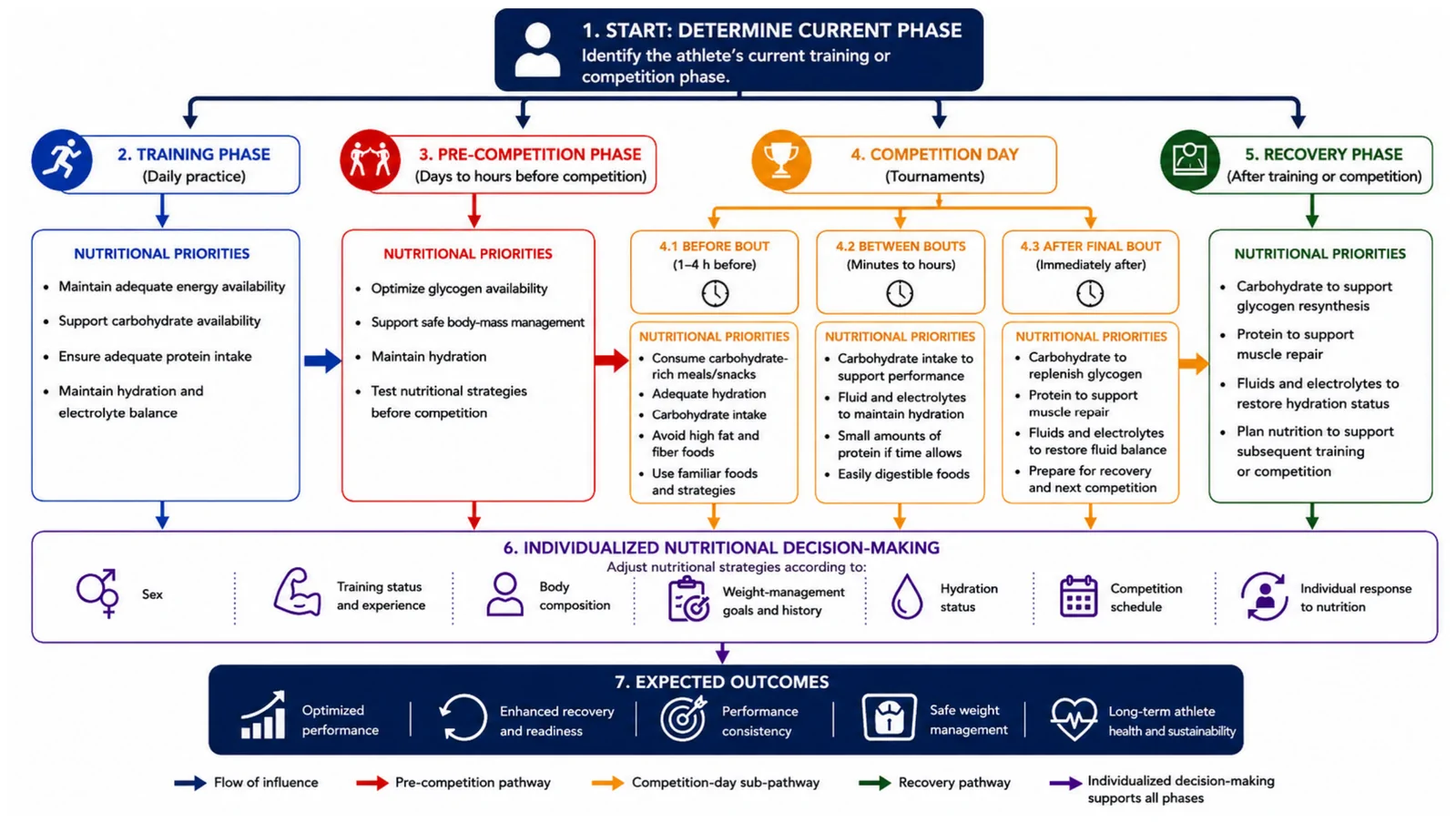
Evidence-Informed Practical Nutritional Decision-Support Tool for Taekwondo. Note. This framework represents an evidence-informed practical decision-support tool based on the best available evidence and should not be interpreted as a validated taekwondo-specific clinical guideline.

Figure 2 summarizes these considerations as an evidence-informed practical decision-support tool for adapting nutritional strategies to training, competition, recovery, and weight-management contexts. It should not be interpreted as a validated taekwondo-specific clinical guideline. Because direct taekwondo-specific intervention evidence remains limited, particularly regarding nutrient dose, timing, formulation, and between-bout recovery strategies, these recommendations should be applied cautiously and individualized to the athlete and competitive context.

## 13. Conclusions

This narrative review provides an evidence-informed synthesis of nutritional strategies relevant to performance, recovery, and weight management in taekwondo. Overall, nutritional planning should be individualized and aligned with training and competition demands, recovery requirements, and safe body-mass management. Particular attention should be given to maintaining adequate energy availability, supporting recovery, and minimizing aggressive weight-loss and dehydration practices.

However, direct taekwondo-specific evidence remains limited, and many of the current practical recommendations are largely extrapolated from comparable combat sports, broader athletic populations, and established sports-nutrition consensus statements and guidelines. Accordingly, these recommendations should be interpreted as evidence-informed and individualized guidance rather than as fully validated taekwondo-specific protocols. Future research should include well-controlled, sport-specific experimental studies to strengthen the evidence base and refine nutritional recommendations for taekwondo athletes.

## 14. Limitations

Although this narrative review provides an integrative synthesis of the available evidence, several limitations should be acknowledged. First, the current body of taekwondo-specific nutrition research remains relatively limited. Consequently, many of the practical recommendations presented in this review are extrapolated from evidence obtained in other athletic populations, including endurance, team, strength, and combat sports with similar physiological demands. Although these recommendations are supported by established sports nutrition principles and have been interpreted within the specific context of taekwondo, their direct applicability to taekwondo athletes requires further sport-specific investigation. Future well-designed experimental studies are needed to establish taekwondo-specific nutritional guidelines, strengthen the sport-specific evidence base for the evidence-informed framework presented in this review, and refine evidence-based recommendations across different competitive levels, sexes, and weight categories.

## Figures and Tables

**Table 1:** Sources and Directness of Evidence Supporting Nutritional Recommendations in Taekwondo.

Nutritional Domain	Source of Evidence	Evidence Interpretation	Key Supporting References
**Carbohydrate availability**	Taekwondo-specific evidence supplemented by broader sports-nutrition evidence	Limited direct taekwondo-specific evidence	[41]
**Protein intake**	Taekwondo-specific observational evidence supplemented by broader sports-nutrition evidence	Limited direct taekwondo-specific evidence	[42]
**Hydration and rehydration**	Taekwondo-specific evidence supplemented by broader evidence	Direct taekwondo-specific evidence	[5,43]
**Ergogenic aids**	Taekwondo-specific systematic evidence; directness varies by supplement	Mixed evidence; supplement-specific	[14]
**Nutrition and cognitive performance**	Limited taekwondo-specific evidence supplemented by broader evidence	Limited direct taekwondo-specific evidence	[43]
**Weight management**	Taekwondo-specific evidence supplemented by combat-sport evidence	Direct taekwondo-specific evidence	[5]

## Data Availability

No new data were created or analyzed in this study. Data sharing is not applicable to this article.
